# Hepatic Inflammation Primes Vascular Dysfunction Following Treatment with LPS in a Murine Model of Pediatric Fatty Liver Disease

**DOI:** 10.3390/ijms26146802

**Published:** 2025-07-16

**Authors:** Hong Huang, Robin Shoemaker, Yasir Alsiraj, Margaret Murphy, Troy E. Gibbons, John A. Bauer

**Affiliations:** 1 Division of Pediatric Research, Department of Pediatrics, University of Kentucky, Lexington, KY 40508, USA; hong.huang@uky.edu (H.H.);; 2 Division of Gastroenterology, Department of Pediatrics, University of Kentucky, Lexington, KY 40508, USA

**Keywords:** pediatric fatty liver, hypercholesterolemia, hepatic inflammatory response, endothelial inflammatory mediators, LPS, inflammatory challenge, vascular endothelial dysfunction

## Abstract

Obesity and pediatric fatty liver disease are increasingly prevalent, yet the underlying mechanisms linking these conditions to heightened inflammatory and immune responses remain poorly understood. Using a murine model reflecting early-life obesity and hepatic steatosis, we tested the hypothesis that obesity-driven hepatic inflammation intensifies systemic immune responses and exacerbates vascular dysfunction following innate immune activation. Newly weaned C57BL/6 mice were fed either a high-saturated-fat, high-cholesterol diet (HFD) or a control diet (CD) for four weeks, modeling adolescence in humans. HFD-fed mice exhibited hepatic and splenic enlargement, elevated plasma cholesterol levels, increased activity levels of liver enzymes (alanine and aspartate aminotransferases), and higher plasma serum amyloid A (SAA) concentrations. Following a sublethal dose of lipopolysaccharide (LPS), the expression of hepatic inflammatory genes (VCAM-1 and iNOS) was significantly elevated in HFD-fed mice, indicating an exaggerated local immune response. Mice fed an HFD also showed significant impairment in endothelium-dependent vasorelaxation compared to CD mice and saline-treated controls, while endothelium-independent responses remained intact. These vascular changes occurred in the context of hepatic inflammation, suggesting that early-life diet-induced steatosis sensitizes the vasculature to inflammatory insult. These findings suggest that obesity-driven hepatic inflammation primes exaggerated systemic immune responses to innate immune stimuli, potentially contributing to the vascular dysfunction and variable clinical morbidity observed in pediatric inflammatory conditions.

## 1. Introduction

The global rise in pediatric hyper-nutrition and related obesity has been paralleled by an increase in metabolic disorders, including fatty liver disease (FLD), which now affects 10% or more of children and adolescents in developed countries [1]. These conditions, characterized by intrahepatic fat accumulation, hepatic cell immune activation, injury, and dysfunction, are now recognized as a leading cause of the need for liver transplant in younger and non-alcoholic patients [2]. The low-level and chronic inflammation associated with FLD has also been linked to long-term cardiovascular disease risks in adults [3]. In light of its growing prevalence in children, and its potentially severe health impact, there is a need to better define pediatric FLD mechanisms and to enhance the understanding of interactions with other obesity-related morbidities in early life.

The relationships between pediatric FLD and adiposity are complex, and in particular, the impact of chronic low-level inflammation in early life on modulating susceptibility to secondary acute immune challenges is poorly understood. This complexity is supported by recent clinical studies demonstrating that pediatric obesity may have a protective or U-shaped effect on mortality in some cases of critical illness [4,5,6], whereas chronic liver disease in children is strongly associated with severe outcomes and/or death [7]. A specific clinical condition where the interactions among hepatic and immune–inflammatory pathways may be particularly relevant is sepsis [8], a condition where prognosis and adverse outcomes in pediatric populations can be extremely unpredictable [9]. A significant number of sepsis cases result from Gram-negative bacterial-derived endotoxin, which evokes intensive inflammatory responses through the toll-like receptor-4 (TLR4) signaling pathway [10]. Its effects can include the induction of acute-phase proteins and the release of cytokines, as well as the activation of endothelial cells and the up-regulation of vascular adhesion molecules [11]. Cholesterol has been shown to mediate endothelial cell activation, contributing to endothelial dysfunction and vascular injury [12,13]. Despite increasing interest in the connection between diet, inflammation, and vascular risk, few studies have directly explored the early hepatic immune response and its temporal relationship with downstream vascular changes in pediatric settings. Our study begins to fill this gap by evaluating liver inflammatory gene expression shortly after LPS challenge in high-fat-fed mice, prior to overt vascular dysfunction.

Taken together, these prior studies suggest a potential link between diet-induced hepatic inflammation and the immune sensitization of the vasculature, particularly the TLR4-mediated mechanism linking cholesterol diet-mediated hepatic inflammation and immune activation with vascular dysfunction. This pathway may have significant relevance to sepsis, where vasculopathy is a major contributor to adverse outcomes [14]. The impact of such interactions in pediatric populations, wherein diet-induced hepatic inflammation coexists with dynamic vascular and immune development, has not been fully elucidated.

In this preclinical study, we utilized a murine model of early-life hepatic steatosis and hypercholesterolemia to investigate whether diet-induced hepatic inflammation sensitizes the vasculature to dysfunction following an innate immune challenge. Our focus was on vascular function outcomes as a readout of systemic response to inflammation in the setting of pediatric fatty liver disease. A further understanding of these interactions and mechanistic pathways may lead to newer strategies to identify patients at risk and/or the use of novel strategies to improve outcomes.

## 2. Results

### 2.1. Young Mice Fed a High-Saturated-Fat and High-Cholesterol Diet (HFD) Exhibit Characteristics of Pediatric Fatty Liver Disease

**Figure 1:**
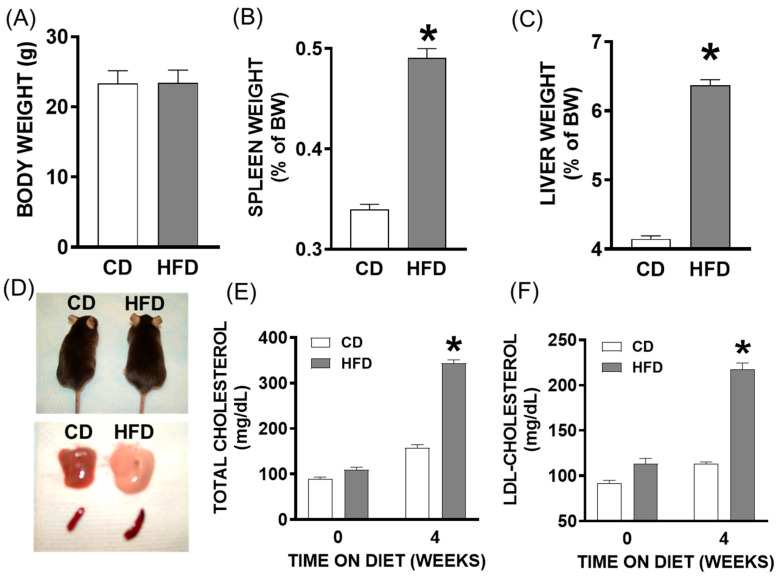
Young mice fed a high-saturated-fat and high-cholesterol diet (HFD) exhibit characteristics of pediatric fatty liver disease. (**A**) Body weight in grams, (**B**) spleen weight and (**C**) liver weight (as percentage of body weight) of young male mice fed an HFD or control diet (CD) for 4 weeks. (**D**) Representative images showing body weight (**Top**) with respective liver and spleen to indicate hepato- and splenomegaly (**Bottom**). (**E**) Total and (**F**) LDL cholesterol levels at zero and four weeks of diet feeding. *, *p* < 0.05 compared to the CD. Data are presented as the mean ± SEM in 25 mice per group.

Newly weaned male C57Bl/6 mice (age 3–4 weeks) were fed a high-saturated-fat/high-cholesterol diet (HFD) or control diet (CD) for four weeks (equivalent in human life to teenage years [15]) to promote liver steatosis. Shown in Figure 1 are characteristics of this murine model with respect to the HFD vs. CD at 4 weeks. Mice fed an HFD for four weeks exhibited no significant difference in body weight compared to mice on a control diet (CD) (Figure 1A). However, HFD mice showed a significant increase in spleen and liver weights relative to CD-fed mice (Figure 1A,C,D; *p* < 0.001) where evidence of lipids in the liver of HFD mice could be observed (Figure 1E). Additionally, after four weeks of diet feeding, total cholesterol concentrations in the plasma of HFD mice were three-fold higher compared to both CD group and baseline levels (Figure 1F; *p* < 0.05). Similarly, LDL cholesterol levels were two-fold elevated in HFD mice compared to the CD group and compared to a subset of control mice and compared to pre-diet feeding (Figure 1F; *p* < 0.05).

### 2.2. Elevated Levels of Hepatic Enzymes and Inflammatory Mediators in Blood and Hepatic Inflammatory Response in Young Mice Fed a High-Fat Diet for Four Weeks

**Figure 2:**
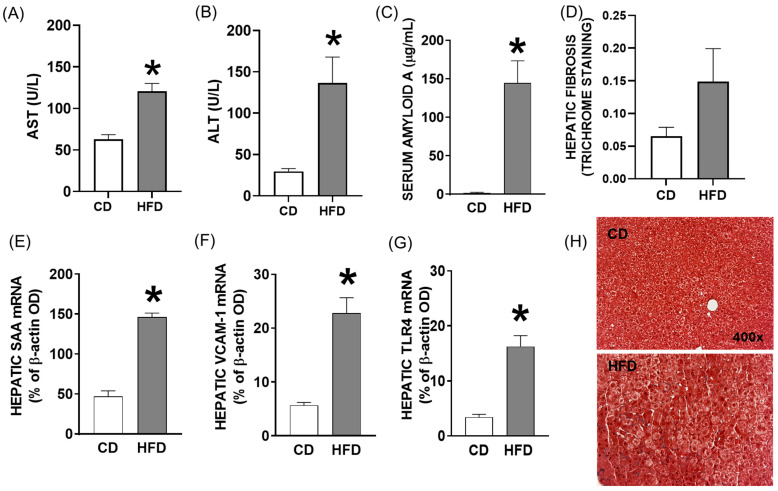
Elevated levels of liver enzymes and inflammatory mediators in young mice fed high-saturated-fat and -cholesterol diet (HFD) and control diet (CD) after four weeks of diet feeding. (**A**,**B**) AST and ALT enzymes and (**C**) SAA concentrations in plasma of HFD-fed versus CD-fed mice. (**D**) Representative images of liver sections from HFD or CD mice (**Top**) with Masson’s trichrome staining for fibrosis (**Bottom**). (**E**) *SAA*, (**F**) *VCAM-1*, and (**G**) *TLR4* mRNA abundance expressed relative to control *β-actin* optimal density (OD) in liver tissue of CD and HFD mice, and (**H**) representative Masson’s trichrome-stained liver sections from CD and HFD mice (400× magnification). *, *p* < 0.01 compared to CD. Data are presented as mean ± SEM in 5 mice per group.

Shown in Figure 2 are features of the HFD vs. CD with respect to hepatic inflammatory status and injury. The baseline effects of the HFD on liver function and inflammation were quantified in mice fed either an HFD or CD for four weeks prior to LPS treatment. The plasma levels of alanine and aspartate aminotransferases (AST and ALT) were significantly increased in HFD compared to CD mice, as were the plasma concentrations of serum amyloid A (SAA), an acute-phase protein produced from the liver during inflammatory response (Figure 2A–C; *p* < 0.01). Moreover, the hepatic mRNA abundance of inflammatory regulating genes, *SAA*, vascular cell adhesion molecule *(VCAM)-1* (a marker of endothelial cell activation), and toll-like receptor *(TLR)-4* (a receptor for LPS and key activator of the innate immune response) was significantly increased in HFD-fed mice (Figure 2E–G; *p* < 0.01). A histological evaluation of liver sections revealed increased lipid accumulation and mild pericentral fibrosis in HFD mice (Figure 2D,H; *p* = 0.06).

### 2.3. Pre-Exposure to High-Fat Diet in Young Mice Primes Hepatic Immune Response to LPS, Potentially Contributing to Vascular Dysfunction

**Figure 3:**
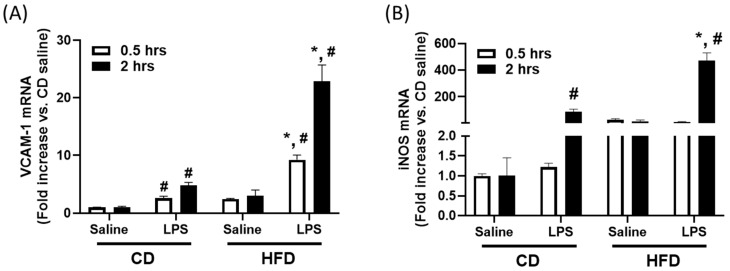
Enhanced hepatic immune response to LPS in high-fat, high-cholesterol diet (HFD)-fed mice. (**A**) VCAM-1 and (**B**) iNOS mRNA abundance in liver tissue from CD and HFD mice at 0.5 and 2 h following treatment with LPS (0.5 mg/kg body weight, intraperitoneal) or saline. *, *p* < 0.05 vs. CD + LPS at the same timepoint. #, *p* < 0.05 vs. saline within the same diet group and at the same timepoint. Data are expressed as the mean ± SEM; *n* = 5 mice per group.

To determine whether a high-fat diet sensitizes the liver to innate immune activation, we measured the hepatic mRNA expression of VCAM-1 and iNOS at early timepoints after LPS administration. Compared to CD-fed mice, HFD-fed mice showed significantly elevated hepatic VCAM-1 mRNA expression at both 0.5 and 2 h post-LPS (Figure 3A; *p* < 0.05). Similarly, iNOS mRNA levels were markedly higher in the livers of HFD-fed mice at 2 h post-LPS (Figure 3B; *p* < 0.05). LPS also induced modest increases in VCAM-1 and iNOS expression in CD-fed mice relative to saline, but the response was substantially amplified in HFD-fed animals. These findings indicate that short-term exposure to a high-fat, high-cholesterol diet primes the hepatic immune environment to respond more robustly to LPS, a response that may underlie the vascular dysfunction observed later in this model.

### 2.4. Impaired Vascular Function After Nonlethal LPS Challenge in Young Mice Fed a High-Fat Diet

**Figure 4:**
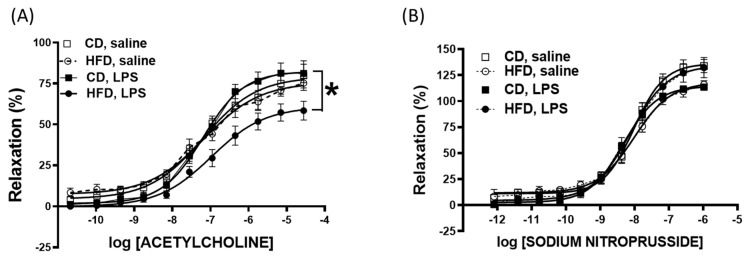
Impaired endothelium-dependent vascular function after LPS challenge in a mouse model of pediatric fatty liver disease. (**A**) Acetylcholine-induced vasorelaxation. (**B**) Sodium nitroprusside-induced vasorelaxation of aortic segments isolated from HFD and CD mice after 24 h of LPS treatment. *, *p* < 0.05 compared to CD treated with LPS. Data are presented as mean ± SEM in *n* = 7 mice per saline-treated diet group and *n* = 8 mice per LPS-treated diet group.

Shown in Figure 4 are isolated vascular responses from animals fed an HFD vs. CD with vs. without in vivo LPS stimulation (assessments 24 hr post-LPS using isolated vascular segments in vitro). To determine the combined effects of LPS and the HFD on vascular endothelial function, we conducted a detailed analysis of the acetylcholine- and sodium nitroprusside-induced vasodilation of aortic segments isolated from HFD and CD mice after 24 h of LPS treatment. Our findings indicated that neither LPS treatment nor the HFD alone significantly altered the acetylcholine-induced vasorelaxant response. However, a notable impairment in vascular relaxation was observed in LPS-treated HFD mice compared to the CD and saline groups (acetylcholine vasorelaxant response: CD—79.1%, LPS—82.4%, HFD—75.6%, and LPS + HFD—60.8%; Figure 4A; *p* < 0.05). This significant reduction in the LPS + HFD group suggests a synergistic negative impact on endothelial function when both inflammatory and dietary stressors are present. In contrast to the endothelium-dependent acetylcholine response, the endothelium-independent vasodilation induced by sodium nitroprusside remained unaffected by either LPS treatment or dietary conditions (Figure 4B). This indicates that the impaired vasorelaxation observed in the LPS + HFD group is specifically related to endothelial dysfunction rather than a general impairment in vascular smooth muscle function.

## 3. Discussion

Obesity and fatty liver disease are closely linked health concerns in children, and both conditions have been associated with alterations in inflammatory and other immune responses. In this study, we utilized a mouse model of pediatric diet-induced fatty liver disease to investigate the interplay between hepatic inflammation and susceptibility to vascular dysfunction following an innate immune challenge. This relatively convenient murine model (e.g., young mice fed a high-fat and high-cholesterol diet for 4 weeks) recapitulated many or most of the clinical presentations of FLD in children with obesity, including hepatic steatosis, hepato- and splenomegaly, hypercholesterolemia, elevated levels of liver enzymes in blood, and hepatic fibrosis compared to control-fed mice. Given its convenience, this animal preparation may be particularly useful for additional preclinical investigations of mechanisms and intervention studies.

Previous studies demonstrated the cholesterol-mediated activation of innate immune pathways in murine models of a high-fat and atherogenic diet [16,17,18,19], while our study confirms and extends these findings to young mice. The current study builds upon prior work showing that diet-induced hepatic inflammation can prime systemic immune responses. We now show that HFD-fed mice exhibit elevated hepatic expression levels of VCAM-1 and iNOS mRNA as early as 0.5–2 h after LPS challenge, suggesting rapid hepatic immune activation in response to innate stimuli. These early hepatic changes may precede and contribute to downstream endothelial impairment.

Since the HFD stimulated vascular inflammatory mediators such as VCAM-1 and iNOS, we determined whether this had effects on the vascular responses of isolated aortic rings. VCAM-1 promotes leukocyte adhesion and endothelial activation, while iNOS contributes to oxidative stress and impaired vasodilation, and both are established contributors to endothelial dysfunction. Isolated vascular responses from HFD-fed mice 24 h after exposure to LPS exhibited selective impaired endothelium-dependent vasorelaxation compared to CD mice. Thus, the hepatic and systemic inflammatory stress related to diet and LPS acute exposure apparently sensitized vascular endothelial dysfunction. Studies in adult patients with fatty liver disease, particularly non-alcoholic fatty liver disease (NAFLD), have also shown that endothelial function is often impaired [20]. This may be an early component of cardiovascular disease risks in pediatric settings [21]. Evidence from other animal studies shows that cholesterol also initiates inflammatory responses in the vasculature and that the cholesterol-mediated activation of endothelial cells can lead to oxidative stress, endothelial dysfunction, and vascular injury [12,13], as shown in a study of children and adolescents with familial hypercholesterolemia [22]. Further studies in this animal model to investigate the vascular endothelial mechanisms of FLD or perhaps clinical studies in children to address these mechanistic consequences appear warranted. Pediatric fatty liver disease has also been associated with severe outcomes in sepsis and other settings of acute infection. These findings are consistent with previous research linking TLR4 activation by LPS to vascular dysfunction and highlighting the role of cholesterol in exacerbating these effects [23,24,25]. Our study is the first to directly link hepatic immune priming with subsequent vascular dysfunction in a pediatric fatty liver disease model.

The findings from this relevant animal model have important implications for understanding the intersection of diet and inflammation in pediatric health. Given the increasing prevalence of high-cholesterol diets and total cholesterol levels in children [26,27], our data suggest that such dietary habits could predispose individuals to enhanced inflammatory responses and vascular dysfunction, particularly in the context of infections or inflammatory conditions such as sepsis. The amplified immune response and endothelial impairment observed in our model could provide insights into the mechanisms linking diet-induced hypercholesterolemia to the increased susceptibility and severity of inflammatory diseases in children. Studies have shown that dietary interventions can significantly impact inflammatory and vascular health in pediatric populations, emphasizing the need for dietary management to prevent such conditions.

This study has several limitations. First, although the mouse model replicates key features of pediatric fatty liver disease, the 4-week dietary exposure may not fully reflect the chronic progression of the disease in children. Second, we did not quantify daily food intake, which limits the interpretation of the similar body weights observed between diet groups. The lack of weight gain in HFD-fed mice may reflect the lower volume consumption of the more calorie-dense HFD. Third, we did not assess body composition, including fat pad weight or muscle mass, which would have helped clarify whether body weight similarity was masking changes in adiposity or lean mass. Fourth, hepatic mRNA expression was assessed using traditional PCR, which is semi-quantitative; while this was performed within the linear range, future studies should confirm the findings using real-time PCR for more accurate quantification. Fifth, we used only male mice to reduce variability, but future studies should evaluate potential sex differences. Lastly, while our findings support a link between hepatic inflammation and vascular dysfunction, additional mechanistic studies are needed to define causal pathways and the temporal relationships between hepatic and vascular immune activation.

## 4. Materials and Methods

### 4.1. Animals and Study Design

This study was approved by the University Animal Care and Use Committee. Male C57BL/6J mice (Jackson Laboratories, Bar Harbor, ME, USA), 3–4 weeks of age (*n* = 100), were fed with a high-fat/high-cholesterol diet or control diet (HFD: 1.25% cholesterol and 16% kcal from fat; CD: 0.03% cholesterol and 5% kcal from fat; Research Diets D12336 and D12337, New Brunswick, NJ, USA) for 0 weeks or 4 weeks (*n* = 25 mice per group) to assess the effects of diet on baseline liver and inflammatory parameters. The HFD (D12336) provided approximately 4.1 kcal/g, and the CD (D12337) provided approximately 3.8 kcal/g. Animals had ad libitum access to both food and water. Food intake was not quantified, which is noted as a limitation in the Discussion. An additional group of *n* = 40 mice fed the CD or HFD for 4 weeks was administered a single intraperitoneal injection of E. coli LPS, serotype 0111:B4 (Sigma-Aldrich, Inc., St. Louis, MO, USA), in sterile saline solution (or saline) at a dose of 0.5 mg/kg body weight [16]. The animals were sacrificed at 0.5 or 2 h post-saline or -LPS treatment. Per timepoint, there were *n* = 5 mice per diet and treatment group. Plasma and flash-frozen livers were stored at −80 °C. Aortas were isolated from mice (*n* = 7–8 per group) 24 h after a single intraperitoneal injection of LPS (0.5 mg/kg body weight) or saline control for the assessment of vascular function, further described below. Cages were assigned by the investigators to diet and treatment groups in order to standardize baseline body weight across groups. All data from all animals were included in the analysis.

### 4.2. Plasma Measurements

Total and low-density lipoprotein (LDL) cholesterol levels were measured by commercial assays (Infinity Cholesterol Reagents (Sigma-Aldrich, Inc., St. Louis, MO). Commercial ELISA kits were used for the quantification of serum amyloid A (SAA; BioSource International, Camarillo, CA, USA). Plasma alanine aminotransferase (ALT) and aspartate aminotransferase (AST) were measured using reagents from Thermo Trace (Louisville, CO, USA) with a kinetic assay [28].

### 4.3. Quantification of Fibrosis in Liver

Liver fibrosis was evaluated in formalin-fixed, paraffin-embedded tissues using Masson’s trichrome stain (Sigma-Aldrich, Inc., St. Louis, MO, USA). Images were captured on a standard upright microscope with a 20× objective (Olympus, Melville, NY, USA) using a digital camera (Diagnostic Instruments, Sterling Heights, MI, USA). Five images were captured per section from 5 CD and HFD mice. Blue collagen staining was defined, and the extent of fibrosis was calculated as the percentage of total tissue area in each image using Image Pro Plus image analysis software version 7 (Media Cybernetics, Silver Springs, MD, USA).

### 4.4. Tissue mRNA Extraction and Quantitative Real-Time Polymerase Chain Reaction (RT-PCR)

Trizol reagent (1 mL) was used to extract total cellular RNA from liver tissue (50 mg) which was reverse-transcribed into cDNA and amplified by PCR using the following primers: vascular cell adhesion molecule-1 (VCAM-1), NM_011693, sense: 5′-GCG CTG TGA CCT GTC TGC AA-3′ and antisense: 5′-GGT GTA CGG CCA TCC ACA G-3′; TLR4, AF185285, sense: 5′-GCT TAC ACC ACC TCT CAA ACT TGA T-3′ and antisense: 5′-ATT ACC TCT TAG AGT CAG TTC ATG G-3′; β-actin, X03672, sense: 5′-ATG GAT GAC GAT ATC GCT-3′ and antisense: 5′-ATG AGG TAG TCT GCT AGG T-3′; inducible nitric oxide synthase (iNOS), NM_010927, sense: 5′-CCT GGA CAA GCT GCA TAT GA-3′ and antisense: 5′-GCT GTG TGG TGG TCC ATG AT-3′ (Invitrogen, Grand Island, NY, USA). PCR products were separated and visualized on a 1.0% agarose ethidium bromide-stained gel. Band intensity was assessed using imaging software (UVP-Labwork version 5.8, now Analytik Jena, Upland CA, USA), and the target mRNA signal was normalized to the β-actin signal in each sample. All reactions were conducted within the linear range of amplification, and gel loading was standardized and conducted in a blinded fashion across all samples.

### 4.5. Isolated Vascular Function in Isolated Aortic Ring Segments

At sacrifice, the thoracic aortas from individual young mice were carefully isolated, using methodologies we have previously published [29]. Segments of aortas (2–3 mm) were equilibrated in Krebs solution at 37 °C bubbled with a mixture of 95% O_2_ and 5% CO_2_. The arterial smooth muscle tone was measured isometrically by a force transducer (Micro-Med, Inc., Louisville, KY, USA). Endothelial-dependent relaxation was assessed by adding acetylcholine (2 × 10^−11^ to 2.8 × 10^−5^ M) to phenylephrine (1.9 μM)-precontracted tissue. The relaxations of vascular rings were calculated as the percentages of the pre-constricted tension and expressed in a cumulative fashion.

### 4.6. Statistical Analyses

All data are graphically represented as the mean ± SEM. GraphPad Prism software version 10.2.3 (Boston, MA, USA) was used to carry out the statistical analysis. Data were tested for normality, and parametric tests were subsequently used. Between-group analysis was performed using an unpaired Student’s *t*-test to test the effects of diet (CD versus HFD) on body and tissue weight, the quantified abundance of mRNA in tissue, plasma markers, and trichrome staining. A two-way analysis of variance was conducted at each timepoint or dose to test the effects of diet and treatment (LPS or saline). Significance was defined as *p* < 0.05.

## 5. Conclusions

In conclusion, our study demonstrated that in a murine model, a high-fat/high-cholesterol diet induces a low-grade inflammatory state and primes the immune system for exaggerated responses to inflammatory stimuli, leading to impaired endothelial function following an innate immune challenge. These results emphasize the need for dietary management to prevent inflammatory and cardiovascular complications, particularly in the pediatric population. Importantly, this vascular dysfunction occurred even in the absence of significant weight gain, highlighting the role of diet quality rather than obesity alone. These insights have important implications for pediatric health, particularly in the context of infections or inflammatory stress. Future research should focus on elucidating the molecular mechanisms underlying these effects and developing targeted interventions to mitigate the health risks associated with high-cholesterol diets and inflammation.

## Data Availability

All data will be made available upon reasonable request by contacting the corresponding author, J.A.B., at john.bauer@uky.edu.
